# Worldwide Prevalence of Head Lice

**DOI:** 10.3201/eid1409.080368

**Published:** 2008-09

**Authors:** Matthew E. Falagas, Dimitrios K. Matthaiou, Petros I. Rafailidis, George Panos, Georgios Pappas

**Affiliations:** Alfa Institute of Biomedical Sciences, Athens, Greece (M.E. Falagas, D.K. Matthaiou, P.I. Rafailidis, G. Panos, G. Pappas); Henry Dunant Hospital, Athens (M.E. Falagas, P.I. Rafailidis); Tufts University School of Medicine, Boston, Massachusetts, USA (M.E. Falagas); Institute of Continuing Medical Education of Ioannina, Ioannina, Greece (G. Pappas)

**Keywords:** pediculosis, louse, Pediculus humanus capitis, prevalence, children, letter

**To the Editor:** Pediculosis capitis has been well-known since antiquity (*1*). Human infestation can result in psychological frustration for parents and children (*2*); furthermore, preventive and therapeutic practices, such as head shaving and the “no-nit” policy of excluding infected children from school, can also induce social stress.

We sought to synthesize the available evidence regarding the worldwide prevalence of lice infestation in the 21st century by conducting a literature search of PubMed and Scopus databases in which we searched for the term *pediculosis.* We also searched Google for the terms *head lice/pediculosis capitis* and individual country names and evaluated references of the articles and reports retrieved through this search. Eligible studies were archived from January 1, 2000, to January 18, 2008.

We retrieved 55 studies (Technical Appendix). Most studies referred to schoolchildren, but some involved refugees, urban slums, child labor, jails, orphanages, and fishing communities.

Most studies had been conducted in Asia; Turkey was overrepresented. Prevalence varied from 0.7% to 59% and was higher in girls and women. Of the 29 studies, 24 involved schoolchildren; the other studies involved refugee children, child laborers, the general population, street children, jail inmates, and children accompanying their mothers in prison.

In Europe, prevalence varied from 0.48% to 22.4%. However, 1 study reported a much higher annual incidence (37.4%) in England (*3*). A study in the Ukraine showed increasing adult representation in the overall affected population (*4*). Six studies involved schoolchildren; the remaining studies involved refugees, homeless persons, and the general population.

Data from Africa, with the exception of 1 study in South Africa, were derived from Egypt. Prevalence varied from 0% to 58.9% and was higher in females. The study in South Africa (*5*) challenges the generally accepted concept that head lice infestation refers to lower socioeconomic status; of 2 schools, 1 in a low socioeconomic status area, populated by black students only, and the other in a high socioeconomic status area, populated by students of various races, head lice infestation was found only in the second school, solely among white pupils. Of 6 studies in Egypt, 4 involved diverse populations: urban poor preschool children, orphanage children, and the general population.

Most studies in the Americas were conducted in Brazil, although we also found data from the United States, Cuba, and Argentina. Prevalence varied from 3.6% to 61.4% and was higher in females. Of 7 studies, 4 involved populations other than schoolchildren to some extent: urban slum residents, fishing community residents, adolescents and adults sampled randomly from the general population, elderly nursing home residents, and persons living with repeatedly infested children. A recent study in Brazil (*6*) noted that prevalence rates determined by visual inspection are twice that of rates determined by hair analysis.

Only 1 study has been performed in Oceania. This study in Australia reported prevalence of 13% and that girls were more likely to have active infection.

Our review shows that pediculosis capitis is widespread throughout the world and does not discriminate on socioeconomic status grounds. The traditional perception of head lice as a parasitosis exclusively associated with schoolchildren of low socioeconomic status is challenged by some of the reports (Technical Appendix).

Most studies underestimate overall prevalence by assessing it in a specific timeframe; to the contrary, head lice infestation is a dynamic process that can spread hypergeometrically in closed environments such as schools and in the community (*7*). The point-prevalence reported by Heukelbach et al (*8*) may represent a more accurate indicator.

Although socioeconomic status seems to be an indicator of the magnitude of lice infestation, more specific determinants are the dynamic processes of hygienic status and overcrowding. A recent study in Turkey compared 2 neighboring villages with different socioeconomic status. The only factor that was statistically significantly related to pediculosis capitis was size of the household; >6 inhabitants was associated with increased prevalence (*9*).

Another parameter that may indirectly influence overall prevalence and account for the leveling of the prevalence gradient between rich and poor is awareness of head lice and preventive and therapeutic practices. A study in Australia showed that although parents prefer to play a major role in prevention and treatment, they may lack insight into recent advances and dilemmas regarding these measures (*10*).

Variations in reported prevalence were found even in data from the same country. These differences can result from surveys being conducted during different seasons, various examination techniques, reporting of active infestation or presence of nits, and potential introduction of effective pediculicides.

Although head lice account for a substantial number of missed schooldays in children, among others, it is surprising that pediculosis capitis is not monitored and prevalence is not regularly reported. Although we cannot extinguish the parasite, effective monitoring and planning will enable us to limit the prevalence and distribution of this parasitosis.

## Supplementary Material

Technical Appendix Worldwide prevalence of head lice infestation*
