# Supplementary material for: Worldwide Prevalence of Head Lice
Source: Emerg Infect Dis. 2008 Sep;14(9):1493–4. doi: 10.3201/eid1409.080368 (PMC2603110; doi:10.3201/eid1409.080368)
Supplement: Technical Appendix — Worldwide prevalence of head lice infestation* [file 08-0368_Techapp-s1.pdf]

Worldwide Prevalence of Head Lice

Technical Appendix

Table. Worldwide prevalence of head lice infestation*

Country (reference)	Year	Setting	Definition	Incidence
Asia				
China (*1*)	2004	Refugee children	NA	43/303 (14.2%)
India (*2*)	2004	Child laborers in a slum area	NA	72/150 (48%)
India (*3*)	2002	Public primary-school children	NA	156/940 (16.59% overall; 20.42% girls, 13.86% boys)
India (*4*)	2002	Jail inmates	NA	15/225 (6.6%)
Iran (*5*)	2006	Children in 12 public rural primary schools	Detection of nits and/or lice	58/847 (6.85%) (55/407 [13.%] girls, 3/440 [0.7%] boys)
Iran (*6*)	2005	Primary-school children	NA	45/1,200 (3.8%); 2/564 boys, 45/636 girls
Iraq (*7*)	2003	409 children from 2 primary schools in Baghdad with different school environment and hygienic status	NA	48.9% incidence in the school with lower school environment and hygiene status. 9.4% in other school
Israel (*8*)	2001	Children 7–10 years of age	Visual examination and combing; detection of nits and/or lice	152/268; (56.7% overall; 61.2% girls, 36.7% boys)
Jordan (*9*)	2000	Elementary public-school children	Detection of nits and/or lice	338/2,519 (13.4% overall; 14.5% girls, 11.1% boys)
South Korea (*10*)	2003	Kindergarten and primary-school children	NA	435/7,495 (5.8% overall; 11.2% girls, 0.9% boys)
South Korea (*11*)	2000	Kindergarten and primary-school children	Detection of nits and/or lice	294/2,288 (12.8% overall; 23.5% girls, 3.9% i boys)
Malaysia (*12*)	2006	11-year-old schoolchildren	Fine-tooth combing and visual examination; detection of nits and/or lice	162/463 (35%)
Nepal (*13*)	2004	A sample of persons 10–39 years of age, street children	NA	16%<br>59%
Nepal (*14*)	2004	Urban schoolchildren	NA	172/ 818 (21%)
Palestine (*15*)	2006	Primary-school girls, 6–14 years of age, from rural and urban area	Detection of nits or lice	340/2,408 (14.1%) with lice<br>843 of 2408 (35%) with nits
Saudi Arabia (*16*)	2006	Urban female schoolchildren from private and preparatory schools	NA	116/2239 (5.2%)
Sri Lanka (*17*)	2001	Children accompanying their mothers in prison	NA	10%
Taiwan (*18*)	2001	Students	NA	615/5121 (12%)
Taiwan (*19*)	2000	Primary-school children (12.9%) from 4 rural districts and 1 urban area	NA	391/3029; More common in rural areas and among girls
Turkey (*20*)	2007	Schoolchildren	Visual inspection	31.1% in a low socioeconomic–status village, 7.7% in a neighboring higher socioeconomic status village (69 and 31 children, respectively)
Turkey (*21*)	2007	Deaf students	NA	6/117 (5.1%)
Turkey (*22*)	2006	Schoolchildren	Visual inspection	9/1134 (0.8%)
Turkey (*23*)	2006	Primary-school children	NA	20/68 (29.4%); 0/32 (0%) boys, 20/36 (55.5%) girls

Country (reference)	Year	Setting	Definition	Incidence
Turkey (*24*)	2006	Primary-school children	Detection of nits and/or lice	117/1261 (9.1%); 16/648 (2.1%) boys, 101/613 (16.4%) girls
Turkey (*25*)	2006	Rural primary-school children	Nits (no adult lice detected)	17/178 (9.5%); 2/104 (1.9%) boys, 15/74 (20.3%) girls
Turkey (*26*)	2005	Schoolchildren 7–14 years of age	Visual examination; detection of nits and/or lice	260/1569 (16.6% overall; 31.8% girls, 2.5% boys)
Turkey (*27*)	2003	Elementary-school children	Detection of nits and/or lice	360/5318 (6.8% overall; 13.3% girls, 1.1% boys)
Turkey (*28*)	2003	Schoolchildren	NA	701/20612 (3.4%)
Turkey (*29*)	2002	Primary-school children	NA	74/785 (9.4%)
Europe				
Albania (*30*)	2002	Refugees from Kosovo (479,223 officially registered)	NA	≈4%
Belgium (*31*)	2005	Schoolchildren 2.5–12 years of age	Wet combing	549/6169 (8.9%)
Belgium (*32*)	2000	Primary-school children in a socially deprived urban area	Visual examination and combing	49/224 (21.9%)
Czech Republic (*33*)	2006	Schoolchildren 6–15 years of age	Dry-hair combing; detection of live lice or dead nits.	75/531 with lice (14.1%)<br>52/531 with nits (9.8%)
England (*34*)	2003	Primary-school children	NA	438/21556 (2.03%); annual incidence 37.4%
England (*35*)	2003	Diagnosis of pediculosis in the West Midland population from 1993-2000	NA	28.2/1,000 patient years at risk
France (*36*)	2007	Urban primary-school children	Fine-tooth combing. Detection of live lice	112/3345 (3.3%)
France (*37*)	2005	Homeless persons	NA	205/930 (22%)
Kosovo (*38*)	2000	Kosovar refugees upon arrival in the United States	Detection of nits and/or lice	107/1051 (10.2%)
Poland (*39*)	2004	Rural schoolchildren, urban schoolchildren	NA	682/42759 (1.59%)<br>252/52394 (0.48%)
Ukraine (*40*)	2006	Population of Ukraine 1990–2004	NA	Referenced as endemic, no actual data shown. Predominance of children in the total infected population in 1990 roughly equal infestation of adults and children in 2004
Africa				
Egypt (*41*)	2003	Rural inhabitants of all ages	NA	1551/8008 (19.37%)
Egypt (*42*)	2002	Population sample	Visual examination	137/2448 (5.6%)
Egypt (*43*)	2001	Primary-, preparatory-, secondary- school children	NA	384/1772 with head or body lice (21.67% overall; 30.26% girls, 17.7% boys, 18.2:1 head lice:body lice ratio)
Egypt (*44*)	2000	Urban poor preschool children	Visual examination	151/ 256 (58.9%)
Egypt (*45*)	2000	Orphanage children 2–6 years of age	NA	64.1%
Egypt (*46*)	2000	Primary-school children	NA	276/510 (54.1%)
South Africa (*47*)	2003	Primary-school children 6–13 years of age (black and white) from 2 rural schools, 1 with low and 1 with high socioeconomic status	Visual examination followed by hair conditioner and fine-tooth combing if evidence of lice found; detection of nits and/or lice	0/300 (0%) in the school with low socioeconomic status; 15/175 (8.6%) in the other school; all infected children were white
Americas				
Argentina (*48*)	2005	Primary-school children from public and private school	Detection of nits and/or lice	842/1370 (61.4%); 296/678 (44%) boys, 546/692 (79%) girls

Country (reference)	Year	Setting	Definition	Incidence
Brazil (*49*)	2007	98 children, 196 adolescents, 119 adults, 90 elderly nursing home residents	Cut hair analysis and visual inspection	13.3%, 5.6%, 5.4%. and 5.5% respectively, by cut hair analysis. Visual inspection doubled this prevalence in general
Brazil (*50*)	2005	Urban slum residents, fishing community residents	NA	634/1460 (43.4%); 170/605 (28.1%)
Brazil (*51*)	2003	Slum population attending a primary healthcare center	NA	Point prevalence 38.2%
Brazil (*52*)	2002	Children 0–15 years of age at day care centers; public, urban, rural schools	NA	309/884 (35%)
Cuba (*53*)	2000	Persons living with children who repeatedly had pediculosis	NA	40/237 (14.54% overall; 82.5% female)
United States (*54*)	2001	Students	Detection of nits or lice	28/1729 (1.6%) with lice<br>63/1729 (3.6%) with nits without lice
Oceana (Australia) (*55*)	2004	Primary-school children	Hair conditioner and fine-tooth combing; detection of nits and/or lice	239/1838 (13%); girls more likely to have active infection

*NA, not available.

References

1. Fan CK, Liao CW, Wu MS, Hu NY, Su KE. Prevalence of *Pediculus capitis* infestation among school children of Chinese refugees residing in mountanous areas of northern Thailand. Kaohsiung J Med Sci. 2004;20:183–7. PubMed

2. Mallik S, Chaudhuri RN, Biswas R, Biswas B. A study on morbidity pattern of child labourers engaged in different occupations in a slum area of Calcutta. J Indian Med Assoc. 2004;102:198–200, 226. PubMed

3. Khokhar A. A study of pediculosis capitis among primary school children in Delhi. Indian J Med Sci. 2002;56:449–52. PubMed

4. Kuruvila M, Shaikh MI, Kumar P. Pattern of dermatoses among inmates of district prison—Mangalore. Indian J Dermatol Venereol Leprol. 2002;68:16–8. PubMed

5. Nazari M, Fakoorziba MR, Shobeiri F. *Pediculus capitis* infestation according to sex and social factors in Hamedan, Iran. Southeast Asian J Trop Med Public Health. 2006;37(Suppl 3):95–8. PubMed

6. Kamiabi F, Nakhaei FH. Prevalence of pediculosis capitis and determination of risk factors in primary-school children in Kerman. East Mediterr Health J. 2005;11:988–92. PubMed

7. Al-Kubiassy W, Abdul Karim ET. Head lice in pupils of two primary schools in Baghdad. J Bahrain Med Soc. 2003;15:34–8.

8. Mumcuoglu KY, Friger M, Ioffe-Uspensky I, Ben-Ishai F, Miller J. Louse comb versus direct visual examination for the diagnosis of head louse infestations. Pediatr Dermatol. 2001;18:9–12. PubMed DOI: 10.1046/j.1525-1470.2001.018001009.x

9. Amr ZS, Nusier MN. Pediculosis capitis in northern Jordan. Int J Dermatol. 2000;39:919–21. PubMed DOI: 10.1046/j.1365-4362.2000.00088.x

10. Sim S, Lee IY, Lee KJ, et al. A survey on head lice infestation in Korea (2001) and the therapeutic efficacy of oral trimethoprim/sulfamethoxazole adding to lindane shampoo. Korean J Parasitol. 2003;41:57–61. PubMed

11. Ha YC, Heo JM, Kim HJ, et al. Infestation status of head louse and treatment with lindane shampoo in children of primary school and kindergarten in Chinju-shi, Kyongsangnam-do, Korea. Korean J Parasitol. 2000;38:41–3. PubMed

12. Bachok N, Nordin RB, Awang CW, Ibrahim NA, Naing L. Prevalence and associated factors of head lice infestation among primary schoolchildren in Kelantan, Malaysia. Southeast Asian J Trop Med Public Health. 2006;37:536–43. PubMed

13. Poudel SK, Barker SC. Infestation of people with lice in Kathmandu and Pokhara, Nepal. Med Vet Entomol. 2004;18:212–3. PubMed DOI: 10.1111/j.0269-283X.2004.00494.x

14. Shakya SR, Bhandary S, Pokharel PK. Nutritional status and morbidity pattern among governmental primary schoolchildren in the Eastern Nepal. Kathmandu Univ Med J. 2004;2:307–14.

15. Al-Shawa RM. Head louse infestations in Gaza governorates. J Med Entomol. 2006;43:505–7. PubMed DOI: 10.1603/0022-2585(2006)43[505:HLIIGG]2.0.CO;2

16. Al-Saeed WY, Al-Dawood KM, Bukhari IA, Bahnassy AA. Prevalence and pattern of skin disorders among female schoolchildren in Eastern Saudi Arabia. Saudi Med J. 2006;27:227–34. PubMed

17. Senanayake MP, Arachchi JK, Wickremasinghe VP. Children of imprisoned mothers. Ceylon Med J. 2001;46:51–3. PubMed

18. Fan PC, Chung WC, Chen ER. Parasitic infections among the aborigines in Taiwan with special emphasis on Taeniasis asiatica. Kaohsiung J Med Sci. 2001;17:1–15. PubMed

19. Wu YH, Su HY, Hsieh YJ. Survey of infectious skin diseases and skin infestations among primary school students of Taitung County, eastern Taiwan. J Formos Med Assoc. 2000;99:128–34. PubMed

20. Balcioglu IC, Kurt O, Limoncu ME, et al. Rural life, lower socioeconomic status and parasitic infections. Parasitol Int. 2007;56:129–33. PubMed DOI: 10.1016/j.parint.2007.01.005

21. Atambay M, Karaman O, Karaman U, Aycan O, Yologlu S, Daldal N. The frequency of intestinal parasites and head lice among students of the Aksemsettin Primary School for Deaf Students. Turkiye Parazitol Derg. 2007;31:62–5. PubMed

22. Ciftci IH, Karaca S, Dogru O, Cetinkaya Z, Kulac M. Prevalence of pediculosis and scabies in preschool nursery children of Afyon, Turkey. Korean J Parasitol. 2006;44:95–8. PubMed

23. Noyan E, Demir V. Investigation of pediculosis carried out as the special study module No. 74, a part of Ege University Medical Faculty's educational program. Turkiye Parazitol Derg. 2006;30:32–4. PubMed

24. Oguzkaya Artan M, Baykan Z, Koc AN. The prevalence of *Pediculus capitis* in students of eight primary schools in the rural area of the Kayseri province. Turkiye Parazitol Derg. 2006;30:112–4. PubMed

25. Ozcelik S, Degerli S, Aslan A. Investigation of the prevalence of *Pediculus* in Alahaci village primary school students in the Sivas province. Turkiye Parazitol Derg. 2006;30:184–6. PubMed

26. Akisu C, Aksoy U, Delibas SB, Ozkoc S, Sahin S. The prevalence of head lice infestation in school children in Izmir, Turkey. Pediatr Dermatol. 2005;22:372–3. PubMed DOI: 10.1111/j.1525-1470.2005.22423.x

27. Kokturk A, Baz K, Bugdayci R, et al. The prevalence of pediculosis capitis in schoolchildren in Mersin, Turkey. Int J Dermatol. 2003;42:694–8. PubMed DOI: 10.1046/j.1365-4362.2003.01836.x

28. Tanyuksel M, Araz RE, Albay A, Aycicek H. Prevalence and treatment of *Pediculus humanus capitis* with 1% permethrin and 0.4% d-phenothrin in Turkey. Acta Medica (Hradec Kralove). 2003;46:73–5. PubMed

29. Inanir I, Sahin MT, Gunduz K, Dinc G, Turel A, Ozturkcan S. Prevalence of skin conditions in primary school children in Turkey: differences based on socioeconomic factors. Pediatr Dermatol. 2002;19:307–11. PubMed DOI: 10.1046/j.1525-1470.2002.00087.x

30. Kondaj R. Management of refugee crisis in Albania during the 1999 Kosovo conflict. Croat Med J. 2002;43:190–4. PubMed

31. Willems S, Lapeere H, Haedens N, Pasteels I, Naeyaert JM, De MJ. The importance of socio-economic status and individual characteristics on the prevalence of head lice in schoolchildren. Eur J Dermatol. 2005;15:387–92. PubMed

32. De Maeseneer J, Blokland I, Willems S, Vander SR, Meersschaut F. Wet combing versus traditional scalp inspection to detect head lice in schoolchildren: observational study. BMJ. 2000;321:1187–8. PubMed DOI: 10.1136/bmj.321.7270.1187

33. Rupes V, Vlckova J, Mazanek L, Chmela J, Ledvinka J. Pediatric head lice: taxonomy, incidence, resistance, delousing. Epidemiol Mikrobiol Imunol. 2006;55:112–9. PubMed

34. Harris J, Crawshaw JG, Millership S. Incidence and prevalence of head lice in a district health authority area. Commun Dis Public Health. 2003;6:246–9. PubMed

35. Smith S, Smith G, Heatlie H, Bashford J, Ashcroft D, Millson D. Head lice diagnosed in general practice in the West Midlands between 1993 and 2000: a survey using the General Practice Research Database. Commun Dis Public Health. 2003;6:139–43. PubMed

36. Durand R, Millard B, Bouges-Michel C, Bruel C, Bouvresse S, Izri A. Detection of pyrethroid resistance gene in head lice in schoolchildren from Bobigny, France. J Med Entomol. 2007;44:796–8. PubMed DOI: 10.1603/0022-2585(2007)44[796:DOPRGI]2.0.CO;2

37. Brouqui P, Stein A, Dupont HT, et al. Ectoparasitism and vector-borne diseases in 930 homeless people from Marseilles. Medicine (Baltimore). 2005;84:61–8. PubMed DOI: 10.1097/01.md.0000152373.07500.6e

38. Manjrekar RR, Partridge SK, Korman AK, Barwick RS, Juranek DD. Efficacy of 1% permethrin for the treatment of head louse infestations among Kosovar refugees. Mil Med. 2000;165:698–700. PubMed

39. Buczek A, Markowska-Gosik D, Widomska D, Kawa IM. Pediculosis capitis among schoolchildren in urban and rural areas of eastern Poland. Eur J Epidemiol. 2004;19:491–5. PubMed DOI: 10.1023/B:EJEP.0000027347.76908.61

40. Kurhanova I. Lice infestation and lice control remedies in the Ukraine. Ann N Y Acad Sci. 2006;1078:357–60. PubMed DOI: 10.1196/annals.1374.070

41. Abdel-Hafez K, Abdel-Aty MA, Hofny ER. Prevalence of skin diseases in rural areas of Assiut Governorate, Upper Egypt. Int J Dermatol. 2003;42:887–92. PubMed DOI: 10.1046/j.1365-4362.2003.01936.x

42. El-Basheir ZM, Fouad MA. A preliminary pilot survey on head lice, pediculosis in Sharkia Governorate and treatment of lice with natural plant extracts. J Egypt Soc Parasitol. 2002;32:725–36. PubMed

43. Morsy TA, el-Ela RG, Mawla MY, Khalaf SA. The prevalence of lice infesting students of primary, preparatory and secondary schools in Cairo, Egypt. J Egypt Soc Parasitol. 2001;31:43–50. PubMed

44. El Sahn AA, Hassan MH, Ftohy EM, Abou-El Ela NE, Eassa SM. Parasitic infections and maternal awareness of preschool children in Karmouz district, Alexandria. J Egypt Public Health Assoc. 2000;75:1–29. PubMed

45. Morsy TA, el-Ela RG, Morsy AT, Nassar MM, Khalaf SA. Two contagious ectoparasites in orphanage children in Nasr City, Cairo. J Egypt Soc Parasitol. 2000;30:727–34. PubMed

46. Omar AA. Ringworm of the scalp in primary-school children in Alexandria: infection and carriage. East Mediterr Health J. 2000;6:961–7. PubMed

47. Govere JM, Speare R, Durrheim DN. The prevalence of pediculosis in rural South African schoolchildren [cited 2008 Jul 17]. S Afr J Sci. 2003;99:21. Available from http://www.jcu.edu.au/school/phtm/PHTM/hlice/papers/govere-2003.pdf

48. Catala S, Junco L, Vaporaky R. *Pediculus capitis* infestation according to sex and social factors in Argentina. Rev Saude Publica. 2005;39:438–43. PubMed DOI: 10.1590/S0034-89102005000300015

49. Borges R, Silva JJ, Rodrigues RM, Mendes J. Prevalence and monthly distribution of head lice using two diagnostic procedures in several age groups in Uberlandia, State of Minas Gerais, Southeastern Brazil. Rev Soc Bras Med Trop. 2007;40:247–9. PubMed

50. Heukelbach J, Wilcke T, Winter B, Feldmeier H. Epidemiology and morbidity of scabies and pediculosis capitis in resource-poor communities in Brazil. Br J Dermatol. 2005;153:150–6. PubMed DOI: 10.1111/j.1365-2133.2005.06591.x

51. Heukelbach J, van HE, Rump B, Wilcke T, Moura RC, Feldmeier H. Parasitic skin diseases: health care-seeking in a slum in north-east Brazil. Trop Med Int Health. 2003;8:368–73. PubMed DOI: 10.1046/j.1365-3156.2003.01038.x

52. Borges R, Mendes J. Epidemiological aspects of head lice in children attending day care centres, urban and rural schools in Uberlandia, central Brazil. Mem Inst Oswaldo Cruz. 2002;97:189–92. PubMed DOI: 10.1590/S0074-02762002000200007

53. Castex M, Suarez S, de la Cruz AM. Presence of pediculosis in people living with children positive to *Pediculus capitis* (Anoplura: Pediculidae). Rev Cubana Med Trop. 2000;52:225–7. PubMed

54. Williams LK, Reichert A, MacKenzie WR, Hightower AW, Blake PA. Lice, nits, and school policy. Pediatrics. 2001;107:1011–5. PubMed DOI: 10.1542/peds.107.5.1011

55. Counahan M, Andrews R, Buttner P, Byrnes G, Speare R. Head lice prevalence in primary schools in Victoria, Australia. J Paediatr Child Health. 2004;40:616–9. PubMed DOI: 10.1111/j.1440-1754.2004.00486.x
